# Special Issue “Ocular Ischemic Diseases: From Molecular Mechanisms to Therapeutics”

**DOI:** 10.3390/ijms27177742

**Published:** 2026-08-29

**Authors:** Deokho Lee

**Affiliations:** The Korean Institute of Nutrition, Hallym University, Chuncheon 24252, Republic of Korea; deokho.lee01@gmail.com

Ocular ischemia is widely considered to cause vision loss and/or impairments. Various ocular diseases, e.g., diabetic retinopathy (DR), retinopathy of prematurity, retinal vein/artery occlusion, pathologic myopia, age-related macular degeneration (AMD), glaucoma, and ocular ischemic syndrome (OIS), are associated with ocular ischemia [1,2,3,4,5,6].

To develop or find promising preventive or protective treatments for ocular ischemic diseases, the complex molecular mechanisms underlying the pathologies in each ocular ischemic case need to be thoroughly unraveled [7,8,9,10,11,12].

This Special Issue attempts to collect original research and review articles that summarize recent state-of-the-art findings, concentrating on understanding the pathologic molecular signaling pathways in many ocular ischemic diseases and presenting promising novel therapeutic molecular targets for the prevention and treatment of ocular ischemia.

We successfully collected three research articles and two review studies in this Special Issue. In the study by Nica et al. (contribution 1), the neutrophil-to-lymphocyte ratio (NLR) was found to be an interesting systemic inflammatory marker/predictor of optical coherence tomography (OCT)-defined severity in acute central retinal artery occlusion (CRAO), with central macular thickness (CMT) showing complementary structural information. This retrospective study in Romania explored the relationship between systemic inflammatory markers and OCT-based structural damages in subjects with acute non-arteritic (NA) CRAO. As it is one of the first clues to connect those parameters to the extent of retinal injury detected via OCT imaging, this research outcome represents an important novel finding in CRAO.

Yang et al. (contribution 2) used the intravitreal transplantation of retinal progenitor cells to enhance outcome parameters in a rat model of DR induced by streptozotocin treatment. They found therapeutic evidence of the treatment in the model, histologically and functionally, using electroretinography, optomotor response, and contrast sensitivity as well as immunohistochemical analyses. Following this study’s outcome, a cell-based therapy to prevent and/or protect against ischemic retinopathies can be advanced, as well as the existing therapeutic approaches.

Maran et al. (contribution 3) suggested promising effects with tonabersat suppressing retinal inflammation after hypoxia–ischemia in neonatal rats. Perinatal hypoxic–ischemic encephalopathy results from oxygen deprivation around the time of birth and could be related to brain injuries as well as severe disabilities in other tissues including the eye/retina. Tonabersat [13,14,15] is one of the well-known oral connexin43 hemichannel blockers. The research group applied this agent to the damaging ischemic retinas. Although further investigations are required, this research trial to evaluate the efficacy of tonabersat to treat hypoxic–ischemic injuries including the brain and eye is very significant.

Two reviews by Coviltir et al. (contribution 4) and Lee et al. (contribution 5) present important aspects of the development of ischemic retinopathies and suggest promising treatments in glaucoma as well as DR and AMD. Although there are many review papers regarding the development of retinopathies [16,17,18], these two review articles focus more on the ischemic injury aspects of retinal diseases. Based on these review articles, more research studies can be performed to find cures for many retinal diseases.

In conclusion, this Special Issue is a new collection of research and review articles aimed at advancing our understanding of the molecular mechanisms and treatment of ocular ischemic diseases. Although more studies are required to develop novel treatments, we hope that these contributions can be connected to future translational medical research.
